# Effect of COVID-19 lockdowns on quality-of-life and health services access by socio-economic status in Australia

**DOI:** 10.1093/heapro/daae096

**Published:** 2024-08-21

**Authors:** Ying Ru Feng, Ian Li, Ingebjorg Kristoffersen, Bruce K Armstrong, David B Preen

**Affiliations:** School of Population and Global Health, The University of Western Australia, Clifton Street Building, Clifton Street, Nedlands, Western Australia, 6009, Australia; School of Management and Marketing, Curtin University, Kent Street, Bentley, Western Australia, 6102, Australia; UWA Business School, The University of Western Australia, Hackett Drive, Crawley, Western Australia, 6009, Australia; School of Population and Global Health, The University of Western Australia, Clifton Street Building, Clifton Street, Nedlands, Western Australia, 6009, Australia; School of Public Health, University of Sydney, Edward Ford Building, Fisher Road, Camperdown, New South Wales, 2050, Australia; School of Population and Global Health, The University of Western Australia, Clifton Street Building, Clifton Street, Nedlands, Western Australia, 6009, Australia

**Keywords:** COVID-19, health service access, inequality, lockdown, quality of life, socio-economic status

## Abstract

This study examined changes in physical and mental health quality-of-life and health services access before and after the onset of the COVID-19 pandemic among individuals of lower and higher socio-economic status (SES) in Australia. Difference-in-differences and logistic regression models were undertaken using data from the Household, Income and Labour Dynamics in Australia (HILDA) survey and government data on COVID-19 lockdowns between January 2020 and February 2021. Individuals from higher SES reported larger decreases in mental health quality-of-life scores than those from lower SES after the onset of the pandemic. Those from lower SES reported less disruption with any health services (24.2% vs 30.4%; OR = 0.68; *p* < 0.001), specifically dental services (8.2% vs 15.4%; OR = 0.51; *p* < 0.001) and allied health services (5.9% vs 8.5%; OR = 0.60; *p* < 0.001), compared with those from higher SES. Additional days under lockdown were associated with reduced access to all health services (OR = 1.19). Furthermore, long-term health conditions (higher SES: OR = 1.54) and scores indicative of poorer physical (lower SES: OR = 1.17; higher SES: OR = 1.07) and mental health (lower SES: OR = 1.16; higher SES: OR = 1.12) were associated with increased health services disruption. While individuals from higher SES were more likely than those from lower SES to experience greater relative declines in mental health and increased disruption with health services access, individuals with a greater apparent need for health services, regardless of SES, may have faced inequalities in accessing these services during the COVID-19 pandemic.

**Contribution to Health Promotion**

- Contrary to expectations, individuals from a higher socio-economic status (SES) reported greater deteriorations in mental health quality-of-life measures after the onset of the COVID-19 pandemic, compared with those from a lower SES.
- Higher SES was also associated with reduced health services access due to the pandemic.
- Regardless of SES, individuals with long-term health conditions or poorer quality-of-life measures reported greater disruption with health services during the COVID-19 pandemic.
- Findings highlight the need to consider accessibility to health services for those who are more vulnerable in the population when preparing for future pandemics or health crises.

## BACKGROUND

During the COVID-19 pandemic, lockdowns and restrictions were implemented by many governments globally to mitigate the spread and impact of the virus. In Australia, this included the closure of international and interstate borders, cancellation of major events and gatherings, density and capacity limits, closure of non-essential services and businesses and stay-at-home orders enacted at different times during the pandemic. The severity of these lockdowns and restrictions enforced by Australian state and territory government bodies contributed to minimizing case numbers and deaths due to COVID-19 early in the pandemic (Clay-Williams *et al*., 2020).

However, COVID-19 restrictions may have had a particularly negative impact on individuals’ psychological wellbeing, with studies suggesting an association with increased depression, anxiety, stress and other mental health issues (Benke *et al*., 2020; Du *et al*., 2020; Jacques-Aviñó *et al*., 2020; Majumdar *et al*., 2020; Meda *et al.*, 2021; Odriozola-González *et al*., 2020; Tull *et al*., 2020; Andersen *et al*., 2021; Fancourt *et al*., 2021; Fountoulakis *et al*., 2021; Jacob *et al*., 2021; Mary-Krause *et al*., 2021; Wu *et al*., 2021). Australian studies have found that lockdowns were associated with poorer mental wellbeing and psychological distress (Bhoyroo *et al*., 2021; Butterworth *et al*., 2022; Griffiths *et al*., 2022). Additionally, they may have contributed to poorer lifestyle behaviours, including less physical activity and greater consumption of alcohol and unhealthy foods (Bhoyroo *et al*., 2021; Nguyen *et al*., 2021), potentially leading to adverse or long-term health outcomes.

Previous studies have also suggested that the COVID-19 pandemic exacerbated health-related inequalities already persistent between people from lower and higher socio-economic status (SES) in Australia (Australian Institute of Health Welfare, 2016). Bower *et al*. (2023) found that low-income and poorer living conditions, including living in housing with low natural light, noise and major building defects, were associated with loneliness during the pandemic, potentially impacting mental health. Regarding health service utilization, Gao *et al.* (2023) found that while usage of mental health services increased in young people aged between 18 and 25 years during the pandemic, the increase was much smaller in those from low socio-economic areas than high socio-economic areas. Furthermore, while telehealth facilities were introduced to combat the declines in accessing health services (Antonucci *et al*., 2020; Aragona *et al*., 2020; Baum and Schwartz, 2020; Butt *et al*., 2020; Cano-Valderrama *et al*., 2020; Clerici *et al*., 2020; Lazzerini *et al*., 2020; Danagoulian and Wilk, 2022), concerns have been raised about barriers to this service, including limited internet or computer accessibility and poor digital literacy, which are more pertinent in those of lower SES (Nouri *et al*., 2020; Taylor *et al*., 2021).

Health-related inequalities could potentially be further exacerbated by longer durations of lockdown. However, previous studies examining changes due to the pandemic often consider lockdowns as a singular event, universally experienced by all participants (Bhoyroo *et al*., 2021; Butterworth *et al*., 2022). In reality, different regions in Australia were subjected to varying durations of restrictions and lockdowns, yet provides a situation akin to a ‘natural experiment’ to explore how health-related outcomes changed over the course of the pandemic and its subsequent lockdowns. Therefore, this study aimed to examine the effect of the COVID-19 pandemic on physical and mental health quality-of-life measures and health services access, and whether these effects differ according to SES. Such information is vital for understanding the effectiveness of different approaches to the pandemic, as it can inform responses to future health crises.

## METHODS

This cohort study uses longitudinal data from two consecutive waves of the Household, Income and Labour Dynamics in Australia (HILDA) survey: wave 19, which collected data in 2019 (pre-pandemic), and wave 20, which collected data in 2020 (after the onset of the pandemic). Additionally, government data on COVID-19 lockdowns from 25 January 2020 to 21 February 2021 were used.

### HILDA Survey data and study sample

The HILDA Survey is an annual, nationally representative survey of approximately 17 000 Australian participants, collecting information on socio-demographic, economic, health, labour/employment and family life characteristics and factors. Each annual survey comprises of four instruments: Household Form, Household Questionnaire, Person Questionnaire and Self Completion Questionnaire (see Wooden *et al*., 2002 for a detailed description about the HILDA Survey).

Waves 19 and 20 data collection occurred between 30 July 2019 and 9 February 2020, and 3 August 2020 and 21 February 2021, respectively. As wave 20 data collection occurred during the COVID-19 pandemic, the survey also included a specific COVID-19 module on how different aspects of life (e.g. employment, education, finances) were affected by the pandemic. Additionally, wave 20 data collection was conducted primarily using telephone and online interviews, in contrast to waves 19 and prior, which used face-to-face interviews and hard-copy interview forms.

In total, 17 462 and 17 070 participants completed the person questionnaire in waves 19 and 20, respectively. Of these participants, 16 172 completed the person questionnaire in both waves.

### Socio-demographic characteristics and COVID-19 outcomes

Data on socio-demographic characteristics, including age, sex, household type, marital status, highest education achieved, employment and long-term health conditions, were obtained from the Person Questionnaire.

The Socio-Economic Indexes for Areas Index of Relative Socio-economic Disadvantage (IRSD) (Australian Bureau of Statistics, 2018) were obtained from participants’ residential household addresses to identify individuals from lower and higher SES areas. Participants who resided within the lowest quintile of IRSD scores were defined as being of ‘lower SES’. Participants in the above four quintiles of IRSD scores were grouped together and defined as being of ‘higher SES’. In wave 19, there were 3315 (19.0%) participants of lower SES and 14 147 (81.0%) participants of higher SES. In wave 20, there were 3176 (18.6%) and 13 894 (81.4%) participants of lower and higher SES, respectively.

The COVID-19 pandemic-related module of wave 20 survey included questions about whether the pandemic resulted in the following: life changed; life changed for the worse; learnt from home; experienced interruption in studies; increased time spent working from home; decreased work hours; decreased income; employment terminated/redundancy/ceased operation of business; claimed JobKeeper or employer claimed JobKeeper on participants’ behalf and received Coronavirus Supplement from the government. The JobKeeper Payment was introduced during the pandemic by the Australian Government as a wage subsidy program for businesses and employers. The subsidy covered the cost of employees’ wages ($1500 per fortnight per employee) for businesses which faced economic hardship due to COVID-19. This allowed employees to remain employed and continue to earn an income and also maintained the connection between employers and employees. In contrast, the Coronavirus Supplement was a fortnightly payment to financially assist those who were on income support. In particular, this included new and existing recipients of the JobSeeker Payment who were already unemployed prior to the pandemic or made unemployed during the pandemic.

### Physical and mental health quality of life

Physical Component Summary (PCS), Mental Health Index-5 (MHI-5) and Mental Component Summary (MCS) scores were obtained from the 36-Item Short Form Survey (SF-36) (Ware and Sherbourne, 1992) to capture quality-of-life measures pertaining to physical and mental health. The SF-36 was administered as part of the Self Completion Questionnaire. PCS and MCS scores were calculated using Australian population norms (Ware *et al*., 1994; Australian Bureau of Statistics, 1997). Higher PCS scores represented better physical health and higher MHI-5 and MCS scores represented better mental health.

### Health services access

Health services access was measured based on responses from the wave 20 COVID-19 pandemic module. Four types of health service providers were examined: general medical services (doctors, clinics or hospitals), dental services, mental health providers and allied health providers. For each health service, participants were asked whether the respective provider had deferred or cancelled a treatment or appointment with them due to COVID-19. Participants were then asked whether they themselves had deferred or cancelled a treatment or appointment with each of the health service providers due to COVID-19. For each health service, if the participant responded that one or both of the above situations had occurred, it was classified as a disruption with the respective health service.

### Lockdown and restrictions data source

Information on lockdowns and restrictions was obtained through media releases from state and territory government websites, as each Australian state and territory government were independently responsible for deciding and enforcing lockdowns and restrictions. This information was obtained up to 21 February 2021 (i.e. end date for wave 20 data collection). Two lockdown types were broadly identified: heightened restrictions and full lockdowns. Heightened restrictions were periods where non-essential businesses and services were required to close/cease operation and state governments encouraged people to stay at home. Full lockdowns were periods of government-imposed lockdowns and stay-at-home orders, where there were only four permissible reasons to leave home: shopping for essential goods and services, caregiving or compassionate reasons, exercising (for a time-limited period) and work (if it was not possible to work from home). Under these definitions, combined lockdown (in days) was calculated by summing the days under both heightened restrictions and full lockdowns. Days under combined lockdown were used in analyses, as the population engaged in similar social behaviours under both heightened restrictions and full lockdowns (e.g. avoiding unnecessary trips in the community, minimizing face-to-face contact). A summary of the days under each type of lockdown due to COVID-19, by Australian jurisdiction, is presented in Supplementary Table S1.

### Statistical analyses

Weighted socio-demographic characteristics and survey responses were summarized using descriptive statistics. Data were weighted using responding person population weights and self-completion questionnaire responding person population weights, provided by HILDA, in order to make inferences about the Australian population. For each wave, differences between lower and higher SES were tested using regression analyses and Rao-Scott chi-square tests.

We employed a standard difference-in-difference method to evaluate changes in quality-of-life measures (PCS, MHI-5 and MCS) between wave 19 and wave 20 for individuals from lower and higher SES. The mathematical equation for the difference-in-differences model has been presented in Supplementary Material S1. Wave 19 and wave 20 represent HILDA data collection during 2019 (which occurred prior to the onset of the pandemic) and 2020 (which occurred after the onset of the pandemic and associated lockdowns), respectively. In these models, time (level 1) was nested within the individual (level 2) and nested within households (level 3). We included controls for age, sex, household type, marital status, highest education achieved, employment and long-term health conditions in the analyses. In addition, we included COVID-19 pandemic-related covariates which had the potential to affect quality of life, including decreased income, employment terminated/redundancy/ceased operation of business, claimed JobKeeper/employer claimed JobKeeper on participants’ behalf and received Coronavirus Supplement from the government.

Secondarily, we employed logistic regression models to examine how the pandemic affected access to health services for individuals from lower and higher SES. Models were adjusted for the following covariates, including age, sex, household type, marital status, employment, highest education achieved, long-term health conditions and quality-of-life measures. A lagged model approach was undertaken, where covariates were obtained from wave 19 data (collected pre-pandemic) and so could act as explanatory factors to predict the outcome of interest (i.e. health services access). Where significant differences in health services access between SES groups were identified, further logistic regression models were used to examine the association between health services access with length under lockdown and health outcomes, after stratification by SES and adjustment for potential confounders outlined above.

All statistical analyses were conducted using SAS v9.4 and used statistical procedures which accounted for the complex survey design of the HILDA survey. The significance level was set at *p* < 0.05.

### Ethics statement

Ethics approval was obtained from the Human Ethics Committee at The University of Western Australia (2022/ET000217).

## RESULTS

### Socio-demographic characteristics and COVID-19 outcomes

Weighted socio-demographic characteristics and COVID-19 pandemic-related outcomes are summarized in Table 1. Participants of lower SES experienced, on average, fewer days under combined lockdown than those of higher SES. The mean number of days under combined lockdown was 60.7 days for the lower SES group and 65.3 days for the higher SES group (*p* = 0.016).

**Table 1:** Weighted socio-demographic characteristics and COVID-19 outcomes for Wave 19 and Wave 20 HILDA study population

	Wave 19^a^ (Pre-pandemic)			Wave 20^a^ (After the onset of the pandemic)		
	Lower SES *n* = 3,268	Higher SES *n* = 13,911	*p*-value^b^	Lower SES *n* = 2,968	Higher SES *n* = 12,961	*p*-value^b^
	Mean (SD) or %	Mean (SD) or %		Mean (SD) or %	Mean (SD) or %	
**Socio-demographic variables**						
Days under lockdown	–	–	–	60.74 (29.46)	65.32 (35.19)	0.016^*^
Age (years)	46.89 (19.45)	44.93 (18.90)	0.003^*^	46.66 (19.66)	45.36 (18.88)	0.103
Sex			0.428			0.608
Male	48.49	49.29		48.61	49.25	
Female	51.51	50.71		51.39	50.75	
Household type			<0.001^*^			<0.001^*^
Multi-person household	83.94	88.70		83.77	88.72	
Lone person household	16.06	11.30		16.23	11.28	
Marital status			<0.001^*^			<0.001^*^
Married/de facto	51.71	61.84		51.24	62.26	
Single	48.29	38.17		48.76	37.74	
Highest education achieved			<0.001^*^			<0.001^*^
Tertiary or higher education	49.98	62.68		50.02	63.29	
Secondary education or lower	50.02	37.32		49.98	36.71	
Employment			<0.001^*^			<0.001^*^
Employed	50.65	66.58		48.82	63.82	
Unemployed/not in the labour force	49.35	33.42		51.18	36.18	
Long-term health conditions			<0.001^*^			<0.001^*^
1 + health condition	41.13	25.80		40.72	26.69	
No health conditions	58.87	74.20		59.28	73.32	
PCS score	46.36 (11.45)	49.86 (10.31)	<0.001^*^	46.98 (11.92)	50.40 (10.19)	<0.001^*^
MHI-5 score	68.83 (19.54)	72.53 (17.71)	<0.001^*^	68.39 (19.41)	71.06 (18.13)	<0.001^*^
MCS score	45.56 (11.72)	47.57 (10.93)	<0.001^*^	45.07 (12.25)	46.46 (11.31)	0.001^*^
**COVID-19 outcomes** ^c^						
Life changed due to COVID-19	–	–	–	88.09	92.36	<0.001^*^
Change was for the worse^d^	–	–	–	48.58	49.33	0.704
Learnt from home^e^	–	–	–	88.13	91.94	0.294
Experienced interruption in studies^f^	–	–	–	37.53	39.33	0.671
Increased time spent working from home^g^	–	–	–	19.86	36.61	<0.001^*^
Decreased work hours^g^	–	–	–	25.53	25.12	0.822
Decreased income^g^	–	–	–	23.38	23.64	0.866
Employment terminated/redundancy/ceased operation of business^g^	–	–	–	6.91	8.29	0.139
Claimed JobKeeper/employer claimed JobKeeper on behalf	–	–	–	21.50	23.51	0.866
Received Coronavirus Supplement from government	–	–	–	6.88	3.95	0.229
Disruption with any health service provider	–	–	–	24.15	30.41	<0.001^*^
Disruption with general medical services	–	–	–	13.95	13.16	0.446
Disruption with dental services	–	–	–	8.23	15.37	<0.001^*^
Disruption with mental health providers	–	–	–	2.59	2.13	0.200
Disruption with other allied health providers	–	–	–	5.94	8.48	<0.001^*^

^*^ *p* < 0.05.

^a^Data have been weighted using cross-sectional responding person population weights and self-completion questionnaire responding person population weights provided by HILDA.

^b^ *p*-values for differences between lower and higher SES calculated using regression analyses for continuous variables and Rao-Scott chi-square tests for categorical variables.

^c^COVID-19 outcomes are categorical with a yes/no response. Percentages (%) presented in this section report the proportion with a yes response for each of these outcomes.

^d^Question only asked to participants who reported life changed due to COVID-19.

^e^Question only asked to participants who were currently enrolled in secondary school.

^f^Question only asked to participant who undertook tertiary or higher education during the COVID-19 pandemic in 2020.

^g^Question only asked to participants who were employed, self-employed, or owned a business at the start of the COVID-19 pandemic (in March 2020).

Abbreviations: MCS = Mental Component Summary; MHI-5 = Mental Health Inventory-5; PCS = Physical Component Summary; SD = standard deviation; SES = socio-economic status.

From wave 20, a greater proportion from lower SES reported living alone (16.2% vs 11.3%; *p* < 0.001), being single (48.8% vs 37.7; *p* < 0.001), not having achieved tertiary or higher education (50.0% vs 36.7%; *p* < 0.001), being unemployed or not in the labour force (51.2% vs 36.2%; *p* < 0.001) and having a long-term health condition (40.7% vs 26.7%; *p* < 0.001), than those from higher SES. Additionally, the lower SES group had poorer physical and mental health quality of life (PCS: *M* = 47.0; MHI-5: *M* = 68.4; MCS: *M* = 45.1) than their higher SES counterparts (PCS: *M* = 50.4; *p* < 0.001; MHI-5: *M* = 71.1; *p* < 0.001; MCS: *M* = 46.5; *p* = 0.001). These socio-demographic characteristics were similar between waves 19 and 20. However, the lower SES group was marginally older (*M* = 46.9; range: 15–98) than the higher SES group (*M* = 44.9; range: 15–100; *p* = 0.003) in wave 19, whereas there was no significant difference in age between the two SES groups in wave 20.

Between waves 19 and 20, physical health quality of life (represented by PCS score) increased by 1.34% and 1.08% for the lower and higher SES groups, respectively. Conversely, mental health quality of life declined. On average, MHI-5 and MCS scores decreased by 0.64% and 1.08%. For the lower SES group, and 2.03% and 5.26% for the higher SES group, respectively, between waves 19 and 20.

In wave 20, a smaller proportion of individuals from lower SES reported that due to the pandemic, life had changed (88.1% vs 92.4%; *p* < 0.001) and time spent working from home increased (19.9% vs 36.6%; *p* < 0.001) than those from higher SES. Additionally, the proportion experienced disruption with any health services (24.2% vs 30.4%; *p* < 0.001), dental services (8.2% vs 15.4%; *p* < 0.001) and allied health providers (5.9% vs 8.5%; *p* < 0.001), was smaller in the lower SES group than the higher SES group. No significant differences were found regarding disruptions with general medical services (14.0% vs 13.2%; *p* = 0.446) and mental health providers (2.6% vs 2.1%; *p* = 0.200) between individuals of lower and higher SES.

### Effect of COVID-19 on quality of life

Results from the difference-in-differences models for each quality-of-life measure (PCS, MHI-5 and MCS) are presented in Table 2 (and Supplementary Figure S1). Prior to the pandemic (wave 19), lower SES was associated with poorer physical health (β = −1.60; *p* < 0.001) and mental health quality of life (MHI-5: β = −2.77; *p* < 0.001; MCS: β = −1.33; *p* < 0.001).

**Table 2:** Difference-in-differences analyses for physical and mental health quality of life

	Coefficient estimate (β)	SE	*p*-value
**Model 1: PCS score**			
**Intercept**	58.52	0.32	<0.001^*^
**SES group**	−1.60	0.18	<0.001^*^
**Time**	0.21	0.07	0.004^*^
**SES group × Time**	−0.15	0.17	0.392
**Decreased income**	0.12	0.20	0.554
**Employment terminated/redundancy/ceased operation of business**	−0.02	0.31	0.947
**Claimed JobKeeper/employer claimed JobKeeper on behalf**	0.28	0.19	0.136
**Received Coronavirus Supplement from government**	−0.17	0.30	0.575
**Model 2: MHI-5 score**			
**Intercept**	59.11	0.66	<0.001^*^
**SES group**	−2.77	0.38	<0.001^*^
**Time**	−1.71	0.14	<0.001^*^
**SES group × Time**	1.07	0.32	<0.001^*^
**Decreased income**	−0.64	0.42	0.124
**Employment terminated/redundancy/ceased operation of business**	−2.31	0.65	<0.001^*^
**Claimed JobKeeper/employer claimed JobKeeper on behalf**	0.22	0.40	0.573
**Received Coronavirus Supplement from government**	−4.15	0.63	<0.001^*^
**Model 3: MCS score**			
**Intercept**	37.93	0.41	<0.001^*^
**SES group**	−1.33	0.24	<0.001^*^
**Time**	−1.22	0.09	<0.001^*^
**SES group × Time**	0.55	0.21	0.009^*^
**Decreased income**	−0.44	0.26	0.089
**Employment terminated/redundancy/ceased operation of business**	−1.52	0.40	<0.001^*^
**Claimed JobKeeper/employer claimed JobKeeper on behalf**	0.24	0.25	0.324
**Received Coronavirus Supplement from government**	−2.65	0.39	<0.001^*^

^*^ *p* < 0.05.

See Supplementary Material S1 for the mathematical equation on the difference-in-differences models. Models were adjusted for additional covariates including age, sex, household type, marital status, employment, highest education achieved and long-term health conditions.

Abbreviation: MCS = Mental Component Summary; MHI-5 = Mental Health Index-5; PCS = Physical Component Summary; SE = standard error; SES = socio-economic status.

The onset of the pandemic (wave 20; represented by Time in the models) had a positive effect on physical health quality of life, associated with increased PCS scores (β = 0.21; *p* = 0.004). The non-significant interaction coefficient indicates that this effect was not statistically different between lower and higher SES (β = −0.15; *p* = 0.392). However, further estimate analyses found that for lower SES, PCS scores did not change significantly between the two time points (increase in score: 0.06; 95% CI: −0.24, 0.36; *p* = 0.691).

Conversely, overall mental health quality of life (MHI-5: β = −1.71; *p* < 0.001; MCS: β = −1.22; *p* < 0.001) declined after the onset of the pandemic. Interaction coefficients in both the MHI-5 (β = 1.07; *p* < 0.001) and MCS (β = 0.55; *p* = 0.009) models indicate that the effect of the onset of the pandemic was statistically different between the SES groups, with lower SES associated with a reduced decline in MHI-5 and MCS scores (i.e. higher SES was associated with a greater decline in mental health quality of life). Further estimate analyses reported that lower SES was associated with a 0.64 decrease (95% CI: 0.07, 1.21; *p* = 0.027) in MHI-5 score and 1.22 decrease (95% CI: 1.04, 1.39; *p* < 0.001) in MCS score after the onset of the pandemic. In contrast, for higher SES, these decreases in MHI-5 and MCS scores were 1.71 (95% CI: 1.45, 1.98; *p* < 0.001) and 1.22 (95% CI: 1.04, 1.39; *p* < 0.001), respectively.

Furthermore, having employment terminated/redundancy/ceasing operation of business (MHI-5: β = −2.31; *p* < 0.001; MCS: β = −1.52; *p* < 0.001) and receiving Coronavirus Supplement (MHI-5: β = −4.15; *p* < 0.001; MCS: β = −2.65; *p* < 0.001) were significantly associated with decreased mental health quality of life, while the effect of decreased income approached significance (MCS: β = −0.44; *p* = 0.089). However, these factors were not associated with changes in physical health quality of life. Claiming JobKeeper was also not associated with changes in any quality-of-life measures.

### Socio-economic status and health services access

Results from the logistic regression analyses to examine health services access are summarized in Supplementary Table S2. Individuals of lower SES observed 32% decreased odds (OR = 0.68; 95% CI: 0.57, 0.80; *p* < 0.001) of any health services disruption, compared with individuals of higher SES. However, this association was only significant for dental services and allied health providers; lower SES was associated with 49% (OR = 0.51; 95% CI: 0.42, 0.62; *p* < 0.001) and 40% decreased odds (OR = 0.60; 95% CI: 0.49, 0.74; *p* < 0.001) of disruption with these services, respectively, than higher SES. SES was not significantly associated with general medical service (*p* = 0.478) or mental health service access (*p* = 0.272).

### Length of lockdown, health outcomes and health services access

Results from the logistic regression models on health services access, stratified by SES, are summarized in Table 3 (and Supplementary Table S3). Regarding any health services disruption, days under lockdown had a significant effect on both SES groups. Every additional 30 days under lockdown was associated with 19% increased odds of any disruption for both lower (OR = 1.19; 95% CI: 1.05, 1.34; *p* = 0.007) and higher SES (OR = 1.19; 95% CI: 1.13, 1.25; *p* < 0.001). Specifically for dental services, there was a 21% (OR = 1.21; 95% CI: 1.04, 1.41; *p* = 0.015) and 14% (OR = 1.14; 95% CI: 1.08, 1.20; *p* < 0.001) increased likelihood of disruption for every increase of 30 days under lockdown associated with lower and higher SES, respectively. Similarly, for allied health providers, increased lockdown length was associated with 43% increased odds (OR = 1.43; 95% CI: 1.22, 1.68; *p* < 0.001) of disruption for lower SES and 29% increased odds (OR = 1.29; 95% CI: 1.20, 1.40; *p* < 0.001) for higher SES. Lockdown duration was associated with general medical service access only for higher SES, where every additional 30 days under lockdown was associated with 1.08 times greater odds (OR = 1.08; 95% CI: 1.01, 1.14; *p* = 0.016) of disruption. Only for lower SES, additional lockdown duration was associated with 1.33 times greater odds of disruption with mental health providers (OR = 1.33; 95% CI: 1.06, 1.67; *p* = 0.015).

**Table 3:** Logistic regression analyses of health services access for lower and higher SES

	Lower SES				Higher SES			
	OR	95% CI for OR		*p*-value	OR	95% CI for OR		*p*-value
**Disruption with any health service provider** ^a^								
Days under lockdown^b^	1.19	1.05	1.34	0.007^*^	1.19	1.13	1.25	<0.001^*^
Long-term health conditions (ref = none)	1.49	1.00	2.24	0.052	1.54	1.33	1.78	<0.001^*^
PCS score^c^	1.17	1.01	1.36	0.038^*^	1.07	1.00	1.14	0.041^*^
MCS score^d^	1.16	1.04	1.30	0.008^*^	1.12	1.07	1.18	<0.001^*^
**Disruption with general medical services** ^a^								
Days under lockdown^b^	0.94	0.82	1.09	0.429	1.08	1.01	1.14	0.016^*^
Long-term health conditions (ref = none)	1.63	1.05	2.53	0.030^*^	1.47	1.24	1.75	<0.001^*^
PCS score^c^	1.31	1.11	1.54	0.001^*^	1.24	1.14	1.34	<0.001^*^
MCS score^d^	1.18	1.03	1.36	0.019^*^	1.25	1.18	1.34	<0.001^*^
**Disruption with dental services** ^a^								
Days under lockdown^b^	1.21	1.04	1.41	0.015^*^	1.14	1.08	1.20	<0.001^*^
Long-term health conditions (ref = none)	1.24	0.79	1.93	0.349	1.15	0.97	1.36	0.111
PCS score^c^	0.90	0.75	1.07	0.238	0.91	0.84	0.99	0.021^*^
MCS score^d^	1.06	0.90	1.24	0.487	0.98	0.92	1.05	0.541
**Disruption with mental health providers** ^a^								
Days under lockdown^b^	1.33	1.06	1.67	0.015^*^	1.07	0.94	1.21	0.319
Long-term health conditions (ref = none)	1.87	0.86	4.04	0.113	2.71	1.92	3.84	<0.001^*^
PCS score^c^	1.24	0.89	1.74	0.201	1.00	0.85	1.18	0.996
MCS score^d^	1.86	1.38	2.51	<0.001^*^	1.84	1.63	2.09	<0.001^*^
**Disruption with allied health providers** ^a^								
Days under lockdown^b^	1.43	1.22	1.68	<0.001^*^	1.29	1.20	1.40	<0.001^*^
Long-term health conditions (ref = none)	1.59	1.01	2.52	0.048^*^	1.62	1.33	1.96	<0.001^*^
PCS score^c^	1.24	1.03	1.49	0.023^*^	1.28	1.18	1.39	<0.001^*^
MCS score^d^	1.12	0.94	1.33	0.197	1.08	0.99	1.18	0.074

^*^ *p* < 0.05.

^a^Logistic regression models for SES domains/subpopulations (lower SES and higher SES) included length of lockdown, age, sex, household type, marital status, highest education achieved, employment, long-term health conditions, PCS score and MCS score. Covariates included in the model (except for length of lockdown) were obtained from wave 19 (pre-pandemic). Full logistic regression models for the effect of each variable included in the model are presented in Supplementary Table S2.

^b^Incremental increase of 30 additional days of lockdown.

^c^Incremental decrease of 1 SD in PCS score (1 SD = 10.69).

^d^Incremental decrease of 1 SD in MCS score (1 SD = 11.33).

Abbreviations: CI = confidence interval; MCS = Mental Component Summary; OR = odds ratio (adjusted); PCS = Physical Component Summary; ref = reference group; SD = standard deviation; SES = socio-economic status.

Associations between long-term health conditions and any health services access were significant for those of higher SES (OR = 1.54; 95% CI: 1.33, 1.78; *p* < 0.001) and approached significance for those of lower SES (OR = 1.49; 95% CI: 1.00, 2.24; *p* = 0.052). For lower SES, this association was only significant for two health service types, with long-term health conditions associated with 1.63 times (OR = 1.63; 95% CI: 1.05, 2.53; *p* = 0.030) and 1.59 times greater odds (OR = 1.59; 95% CI: 1.01, 2.52; *p* = 0.048) of disruption with general medical services and allied health providers, respectively, compared with no long-term health conditions. Similarly, for higher SES, it was associated with 1.47 times (OR = 1.47; 95% CI: 1.24, 1.75; *p* < 0.001) and 1.62 times greater odds (OR = 1.62; 95% CI: 1.33, 1.96; *p* < 0.001) of disruption with general medical services and allied health providers, respectively. Additionally, long-term health conditions were associated with 2.71 times greater odds (OR = 2.71; 95% CI: 1.92, 3.84; *p* < 0.001) of disruption with mental health providers only for higher SES, compared with no long-term health conditions. No significant associations between long-term health conditions and dental services disruption for either SES group were observed.

Poorer physical health quality of life was significantly associated with any health service disruption for both lower (OR = 1.17; 95% CI: 1.01, 1.36; *p* = 0.038) and higher SES (OR = 1.07; 95% CI: 1.00, 1.14; *p* = 0.041). For lower SES, every 1 SD decrease in PCS score was associated with 1.31 times (OR = 1.31; 95% CI: 1.11, 1.54; *p* = 0.001) and 1.24 times greater odds (OR = 1.24; 95% CI: 1.03, 1.49; *p* = 0.023) of disruption with general medical services and allied health providers, respectively. Similarly, for higher SES, this decrease was associated with 1.24 times (OR = 1.24; 95% CI: 1.14, 1.34; *p* < 0.001) and 1.28 times greater odds (OR = 1.28; 95% CI: 1.18, 1.39; *p* < 0.001) of disruption, respectively, with the same services. However, every decrease in 1 SD in PCS score was associated with 9% decreased odds of disruption with dental services (OR = 0.91; 95% CI: 0.84, 0.99; *p* = 0.021) in those of a higher SES.

Poorer mental health was significantly associated with any health services access for both lower (OR = 1.16; 95% CI: 1.04, 1.30; *p* = 0.008) and higher (OR = 1.12; 95% CI: 1.07, 1.18; *p* < 0.001) SES. For every 1 SD decrease in MCS score in those of a lower SES, odds of disruption increased by 1.18 times (OR = 1.18; 95% CI: 1.03, 1.36; *p* = 0.019) and 1.86 times (OR = 1.86; 95% CI: 1.38, 2.51; *p* < 0.001) with general medical services and mental health providers, respectively. For higher SES, odds of disruption increased by 1.25 times (OR = 1.25; 95% CI: 1.18, 1.34; *p* < 0.001) and 1.84 times (OR = 1.84; 95% CI: 1.63, 2.09; *p* < 0.001), respectively, for the same services. Additionally, the association between poorer mental health and reduced access with allied health providers approached significance for higher SES (OR = 1.08; 95% CI: 0.99, 1.18; *p* = 0.074).

## DISCUSSION

Our study found that while mental health declined in both SES groups after the onset of the COVID-19 pandemic, this decrease was greater in individuals from higher SES. Furthermore, those of higher SES reported a greater likelihood of health services disruption, specifically with dental services and allied health services. Reduced health services access was also associated with exposure to longer lockdown durations, poorer pre-pandemic physical and mental health and long-term health conditions.

Notably, we found a greater decrease in mental health quality-of-life measures following the onset of the pandemic in individuals of higher SES, than those of lower SES. Previous studies reported contrasting results, with authors suggesting that lockdowns tend to disproportionately affect the health outcomes of poorer populations (Pieh *et al*., 2020; Lehmann *et al*., 2021; Myhr *et al*., 2021). One possible explanation for our findings could be that those from higher SES had higher baseline quality-of-life levels prior to the pandemic’s onset and were more likely to be unaccustomed to the adversity it caused. The prior lack of exposure to certain significant life challenges could have led to a sense of ‘life shock’, resulting in more negative perceptions about the social changes brought about by the pandemic. Conversely, individuals from lower SES may have been more familiar with these hardships. For example, individuals from higher SES may have only had their first exposure to food scarcity after the onset of the pandemic, as panic buying was affecting the supply chain and the availability of staple grocery items (Godrich *et al*., 2022). On the other hand, those from lower SES may have been more likely to have prior exposure to food scarcity or limited access to other ‘essential’ items due to financial difficulties. Wanberg *et al*. (2020) found an association between higher income levels and greater declines in life satisfaction due to COVID-19. They suggested that these declines may be due to the pandemic threatening the higher preceding expectations for constant resource availability (Wanberg *et al*., 2020). However, in our study, changes in MCS and MHI-5 scores were small in magnitude for both SES groups. The largest change observed was approximately a 5% decrease in MCS score for higher SES between waves 19 and 20. Further examination of longitudinal trends in quality-of-life measures over multiple waves would be beneficial in examining the isolated effect of the pandemic.

Additionally, COVID-19 employment-related outcomes, including termination of employment, redundancy and ceasing business operations, and potentially decreased income, had significant negative effects on mental health, as expected. While receiving the Coronavirus Supplement was also associated with poorer mental health, claiming JobKeeper was not. Although both provided financial support, a key distinction between the two was that the Coronavirus Supplement benefitted those employed during the pandemic, while JobKeeper benefitted those unemployed or had lost their job during the pandemic. This suggests that financial insecurity (i.e. decreased income) and job insecurity or lack of employment (i.e. termination of employment, redundancy and ceasing business operations, receiving the Coronavirus Supplement) may be prominent driving forces behind declines in mental health in the Australian workforce during the pandemic. Conversely, JobKeeper has the potential to strengthen both financial and job security and, therefore, had no significant effect on mental health. However, Botha *et al*. (2022) observed the opposite, where Coronavirus Supplement was associated with lower financial stress and mental distress. Further investigation should examine the effectiveness of payment subsidies and stimulus packages and its effect on health and quality of life.

Given that lower SES groups generally experience more health-related inequalities (Australian Institute of Health Welfare, 2016), it was interesting that our findings suggested a contrasting trend for accessing health services. Results indicated that higher SES was associated with greater disruption in health services access during the pandemic. A possible reason for this could be that those from lower SES already had low pre-pandemic engagement levels with health services and were not as affected by the pandemic. In contrast, individuals from higher SES, who were more likely to frequent certain health services, were then subsequently more likely to experience disruptions due to the pandemic.

Additionally, there were greater reports of disruption specifically for access to dental and allied health services, which are often considered discretionary (non-urgent) health services. Under Medicare Australia, coverage for these services is limited compared with general medical, hospital and mental health care. While private health insurances may cover costs for dental and allied health services that aren’t covered under Medicare, those living in areas of high socio-economic disadvantage have the lowest levels of private health insurance (Australian Bureau of Statistics, 2017). Prior to the pandemic, individuals from lower SES were less inclined to engage with dental and allied health services, which may be due to the associated out-of-pocket financial costs. A 2018 systematic review found that Australian adults who were dentally insured had more regular access to dental care (Gnanamanickam *et al*., 2018). Similarly, previous reports found that 32% of people from the least disadvantaged areas had consulted with ‘other health professionals (i.e., allied health professionals)’ in the last 12 months, compared with 25% from the most disadvantaged areas (Australian Bureau of Statistics, 2017). Therefore, individuals of higher SES, who were more likely to engage frequently with dental and allied health providers prior to the pandemic, were then more likely to experience greater disruption with accessing these services after the pandemic began, as seen in our study. Additionally, dental services and certain allied health services (e.g. physiotherapy, optometry) often require ‘hands-on’ examination, posing challenges in delivering their services remotely via telehealth (Breton *et al*., 2021). This, combined with increased avoidance of face-to-face contact, may have contributed to greater disruption with these health services.

Longer lockdown durations were also associated with reduced health services access. During the pandemic, governments discouraged non-essential travel within the community, occasionally imposing travel restrictions outside a specified distance from home. In health settings, density limits led to capacity restrictions in waiting rooms and physicians reducing the number of patient appointments. This likely contributed to a backlog of health appointments, which grew with extended lockdown durations. A prominent example includes the backlog of elective surgeries due to all non-urgent and some semi-urgent elective surgeries being suspended in the early stages of the pandemic by the Australian Federal Government. When these restrictions were lifted, waiting times for most scheduled procedures increased (Australian Institute of Health and Welfare, 2022a). Notably, lockdown duration was not associated with accessing general medical services for lower SES and mental health providers for higher SES, suggesting a somewhat successful transition to telehealth. However, the discrepancy in accessing mental health services between the two SES groups is still concerning. Research prior to the pandemic has already highlighted the socio-economic disparities regarding mental health service use in Australian adults (Meadows *et al*., 2015). During the pandemic, mental health became a major focus for Australian government bodies (Australian Institute of Health and Welfare, 2022b), with several initiatives and funds being announced to support the mental health system (National Mental Health Commission, 2021). However, the World Health Organization (WHO) reported that 93% of countries experienced disruptions to mental, neurological and substance use services (World Health Organization, 2020).

Finally, poorer health outcomes, including long-term health conditions and lower physical and mental health, were associated with disruption to health services access. Concerningly, it suggests that individuals who were more likely to require these health services, irrespective SES, were at a higher risk of experiencing service disruptions. This could lead to delays in treatment or care and potentially exacerbate existing health issues. This highlights the issue of health service disparities during the COVID-19 pandemic and suggests the need to prioritize equitable access to health service, especially for vulnerable populations.

A strength of this study was the use of the population-level HILDA data, which allowed us to conduct within-individual analysis covering a large sample. However, a limitation is the potential underrepresentation of individuals with poorer general and mental health and quality of life, as they may be less likely to participate (Korkeila *et al*., 2001; Cheung *et al*., 2017; Perales and Baffour, 2018). Additionally, questions about health service access were not limited to those who had scheduled appointments and may have resulted in conservative estimates, as the proportion of these service disruptions would be underestimated. Furthermore, the study does not account for participants’ digital literacy and internet or computer access, which plays a major role in accessing telehealth services. Exploring this relationship will be pertinent due to the increasing popularity of remote telehealth services. Although we take into consideration the effect of lockdown durations, there was still major variability surrounding the restrictions enforced during the pandemic between geographical regions. Therefore, we were unable to account for participants who may have been experiencing more severe restrictions or lockdowns, which could particularly affect quality-of-life measures. Given the self-reporting nature of the study, it is possible that the findings may have been influenced by differential recall among the study population. However, it is important to note that the recall period was relatively short. Additionally, data collection shifted to telephone interviews in wave 20, while previous waves conducted face-to-face interviews. However, HILDA has reported that wave 20 data were of similar quality to data from previous waves (Watson *et al*., 2021).

## CONCLUSION

Although we observed greater decreases in mental health among individuals of lower and higher SES after the onset of the COVID-19 pandemic, declines were still relatively small. While lockdowns played a crucial role in preventing illnesses and deaths due to COVID-19, they may have had adverse effects on other aspects of health and wellbeing, including mental health and access to health services. Greater disruptions in health services were observed more commonly in those who were more likely to have been using these services prior to the pandemic and also those with a greater apparent need for these services. These included individuals of higher SES, but concerningly also included those with long-term health conditions and poorer physical and mental health. These findings emphasize the importance of considering these issues in decision-making processes when responding to future pandemics. Further research should focus on examining whether more severe restrictions had a greater effect on mental health, telehealth use and barriers to telehealth in different populations in Australia and also evaluate the mechanisms behind how government subsidies and financial support could improve mental wellbeing during periods of health crises.

## Supplementary Material

daae096_suppl_Supplementary_Material

## Data Availability

Data used in this article were obtained from a third party and are not publicly available. Data are available to researchers through the Australian Data Archive (ADA) (https://dataverse.ada.edu.au/).
