# Supplementary material for: Effect of COVID-19 lockdowns on quality-of-life and health services access by socio-economic status in Australia
Source: Health Promot Int. 2024 Aug 21;39(4):daae096. doi: 10.1093/heapro/daae096 (PMC11336672; doi:10.1093/heapro/daae096)
Supplement: daae096_suppl_Supplementary_Material [file daae096_suppl_supplementary_material.docx]

**Supplementary Material 1. Regression Model for Difference-in-Differences**

$$Y= \beta_{0}+\beta_{1}*\left[ SES group \right]+ \beta_{2}*\left[ Time \right]+\beta_{3}*\left[ SES group \times Time \right]+\beta_{4}\left[ Covariate 1 \right]\ldots+\beta_{n}\left[ Covariate x \right]+ \epsilon$$

Where:

- $Y$ is the $outcome$, which is the quality-of-life score (PCS, MHI-5, or MCS score)
- $\beta_{0}$ is the $intercept$, representing the average quality-of-life score prior to the onset of the COVID-19 pandemic
- $\beta_{1}$ is the coefficient for $SES group$, representing the difference in the quality-of-life score between the two SES groups
  - $SES group$ will assume the value of 1 for the lower SES group
  - $SES group$ will assume the value of 0 for the higher SES group
- $\beta_{2}$ is the coefficient for $Time$, representing the difference in the quality-of-life score between wave 19 and wave 20
  - $Time$ will assume the value of 1 for the wave 20 time period (which was after the onset of the pandemic, where data collection for wave 20 occurred)
  - $Time$ will assume the value of 0 for the wave 19 time period (pre-pandemic, where data collection for wave 19 occurred)
- $\beta_{3}$ is the coefficient for the interaction between $SES group$ and $Time$, representing the difference in the quality-of-life score between wave 19 and wave 20 for the lower SES group, relative to the higher SES group
  - $SES group \times Time$ will assume the value of 1 for the wave 20 time period for the lower SES group
  - Elsewhere, $SES group \times Time$ will assume the value of 0
- $\beta_{4}$ to $\beta_{n}$ are the coefficients for $Covariate 1$ to $Covariate x$ adjusted for in the models, including age, sex, household type, marital status, employment, highest education achieved, long-term health conditions, decreased income, employment terminated/redundancy/ceased operation of business, claimed JobKeeper/employer claimed JobKeeper on behalf, received Coronavirus Supplement from government
- $\epsilon$ represents the error term

**Supplementary Table 1. Days of Lockdowns for Area and Regions in Australia prior to 21^st^ February 2021**

| **State** | **Area/Region** | **Heightened Restrictions**  **(Days)** | **Full Lockdown**  **(Days)** | **Combined Lockdown**  **(Days)** |
| --- | --- | --- | --- | --- |
| **New South Wales** | Northern Beaches (Northern Zone) | 8 | 67 | 75 |
|  | Northern Beaches (Southern Zone) | 8 | 60 | 68 |
|  | Greater Sydney (Excluding Northern Beaches) | 8 | 45 | 53 |
|  | Rest of NSW | 8 | 45 | 53 |
| **Victoria** | Postcodes^a^: 3038, 3064, 3047, 3060, 3012, 3032, 3055, 3042, 3021, 3046 | 8 | 183 | 191 |
|  | Postcodes^b^: 3031, 3051 | 8 | 180 | 188 |
|  | Rest of Metropolitan Melbourne (Excluding postcodes^a,b^) | 8 | 177 | 185 |
|  | Mitchell Shire | 8 | 136 | 144 |
|  | Rest of VIC (Excluding Mitchell Shire) | 8 | 107 | 115 |
| **Queensland** | Greater Brisbane | 6 | 36 | 42 |
|  | Rest of QLD (Excluding Greater Brisbane) | 6 | 33 | 39 |
| **South Australia** | All | 49 | 4 | 53 |
| **Western Australia** | Metropolitan Perth, Peel Region, and South West Region | 35 | 5 | 40 |
|  | Rest of WA (Excluding Metropolitan Perth, Peel Region, and South West Region) | 35 | 0 | 35 |
| **Tasmania** | All | 8 | 41 | 49 |
| **Northern Territory** | All | 39 | 0 | 39 |
| **Australian Capital Territory** | All | 39 | 0 | 39 |
| Abbreviations: NSW=New South Wales; QLD=Queensland; VIC=Victoria; WA=Western Australia | | | | |

**Supplementary Table 2. Full logistic regression analyses of health services access**

|  | **OR** | **95% CI for OR** | | **p-value** |
| --- | --- | --- | --- | --- |
| **Disruption with any health service provider^a^** | | | | |
| SES (ref=lower SES) | 0.68 | 0.57 | 0.80 | <0.001* |
| Age (years) | 1.08 | 1.04 | 1.12 | <0.001* |
| Sex (ref=male) | 1.65 | 1.51 | 1.81 | <0.001* |
| Household type (ref=multi-person household) | 1.02 | 0.90 | 1.15 | 0.815 |
| Employment (ref=employed) | 1.08 | 0.96 | 1.21 | 0.186 |
| Highest education achieved  (ref=tertiary or higher education) | 0.73 | 0.66 | 0.81 | <0.001* |
| Long-term health conditions (ref=none) | 0.68 | 0.59 | 0.77 | <0.001* |
| PCS score^b^ | 1.09 | 1.03 | 1.15 | 0.003* |
| MCS score^c^ | 1.13 | 1.08 | 1.19 | <0.001* |
| **Disruption with general medical services^a^** | | | | |
| SES (ref=lower SES) | 0.94 | 0.78 | 1.12 | 0.478 |
| Age (years) | 1.04 | 1.01 | 1.08 | 0.025* |
| Sex (ref=male) | 1.66 | 1.45 | 1.88 | <0.001* |
| Household type (ref=multi-person household) | 1.00 | 0.85 | 1.17 | 0.975 |
| Employment (ref=employed) | 0.87 | 0.76 | 1.01 | 0.070 |
| Highest education achieved  (ref=tertiary or higher education) | 0.85 | 0.75 | 0.97 | 0.012* |
| Long-term health conditions (ref=none) | 0.68 | 0.58 | 0.81 | <0.001* |
| PCS score^b^ | 1.26 | 1.18 | 1.35 | <0.001* |
| MCS score^c^ | 1.22 | 1.15 | 1.29 | <0.001* |
| **Disruption with dental services^a^** | | | | |
| SES (ref=lower SES) | 0.51 | 0.42 | 0.62 | <0.001* |
| Age (years) | 1.11 | 1.06 | 1.15 | <0.001* |
| Sex (ref=male) | 1.52 | 1.37 | 1.69 | <0.001* |
| Household type (ref=multi-person household) | 0.98 | 0.84 | 1.15 | 0.825 |
| Employment (ref=employed) | 1.23 | 1.05 | 1.43 | 0.011* |
| Highest education achieved  (ref=tertiary or higher education) | 0.62 | 0.54 | 0.72 | <0.001* |
| Long-term health conditions (ref=none) | 0.88 | 0.76 | 1.03 | 0.106 |
| PCS score^b^ | 0.90 | 0.84 | 0.97 | 0.005* |
| MCS score^c^ | 1.00 | 0.94 | 1.06 | 0.988 |
| **Disruption with mental health providers^a^** | | | | |
| SES (ref=lower SES) | 0.83 | 0.59 | 1.16 | 0.272 |
| Age (years) | 0.70 | 0.64 | 0.77 | <0.001* |
| Sex (ref=male) | 1.41 | 1.06 | 1.87 | 0.017* |
| Household type (ref=multi-person household) | 1.03 | 0.74 | 1.42 | 0.876 |
| Employment (ref=employed) | 0.67 | 0.51 | 0.87 | 0.003* |
| Highest education achieved  (ref=tertiary or higher education) | 0.86 | 0.64 | 1.15 | 0.297 |
| Long-term health conditions (ref=none) | 0.38 | 0.28 | 0.53 | <0.001* |
| PCS score^b^ | 1.04 | 0.90 | 1.20 | 0.586 |
| MCS score^c^ | 1.84 | 1.65 | 2.06 | <0.001* |
| **Disruption with allied health providers^a^** | | | | |
| SES (ref=lower SES) | 0.60 | 0.49 | 0.74 | <0.001* |
| Age (years) | 1.09 | 1.04 | 1.14 | <0.001* |
| Sex (ref=male) | 1.88 | 1.59 | 2.24 | <0.001* |
| Household type (ref=multi-person household) | 1.00 | 0.84 | 1.20 | 0.961 |
| Employment (ref=employed) | 1.11 | 0.93 | 1.33 | 0.259 |
| Highest education achieved  (ref=tertiary or higher education) | 0.60 | 0.52 | 0.69 | <0.001* |
| Long-term health conditions (ref=none) | 0.64 | 0.53 | 0.76 | <0.001* |
| PCS score^b^ | 1.27 | 1.18 | 1.37 | <0.001* |
| MCS score^c^ | 1.08 | 1.00 | 1.17 | 0.056 |
| ^a^ Logistic regression models included SES, age, sex, household type, employment, highest education achieved, long-term health conditions, PCS score and MCS score. Covariates included in the model were obtained from wave 19 (pre-pandemic).  ^b^ Incremental decrease of one SD in PCS score (1SD=10.69)  ^c^ Incremental decrease of one SD in MCS score (1SD=11.33)  Abbreviations: CI=Confidence interval; OR=Odds ratio (adjusted); MCS=Mental Component Summary; PCS=Physical Component Summary; ref=reference group; SD=Standard deviation; SES=Socio-economic status | | | | |

**Supplementary Table 3. Full logistic regression analyses of health services access for lower and higher SES**

|  | **Lower SES** | | | | **Higher SES** | | | |
| --- | --- | --- | --- | --- | --- | --- | --- | --- |
|  | **OR** | **95% CI for OR** | | **p-value** | **OR** | **95% CI for OR** | | **p-value** |
| **Disruption with any health service provider^a^** | | | | | | | | |
| Days under lockdown^b^ | 1.19 | 1.05 | 1.34 | 0.007* | 1.19 | 1.13 | 1.25 | <0.001* |
| Age (years) | 1.04 | 0.95 | 1.13 | 0.410 | 1.07 | 1.03 | 1.12 | 0.002* |
| Sex (ref=male) | 1.68 | 1.32 | 2.13 | <0.001* | 1.64 | 1.49 | 1.80 | <0.001* |
| Household type (ref=multi-person household) | 0.96 | 0.69 | 1.34 | 0.831 | 1.20 | 1.00 | 1.45 | 0.054 |
| Marital status (ref=married/de facto) | 1.13 | 0.84 | 1.52 | 0.423 | 0.81 | 0.69 | 0.97 | 0.019* |
| Highest education achieved  (ref=tertiary or higher education) | 0.83 | 0.68 | 1.02 | 0.074 | 0.75 | 0.67 | 0.84 | <0.001* |
| Employment (ref=employed) | 1.03 | 0.78 | 1.37 | 0.814 | 0.93 | 0.82 | 1.07 | 0.308 |
| Long-term health conditions (ref=none) | 1.49 | 1.00 | 2.24 | 0.052 | 1.54 | 1.33 | 1.78 | <0.001* |
| PCS score^c^ | 1.17 | 1.01 | 1.36 | 0.038* | 1.07 | 1.00 | 1.14 | 0.041* |
| MCS score^d^ | 1.16 | 1.04 | 1.30 | 0.008* | 1.12 | 1.07 | 1.18 | <0.001* |
| **Disruption with general medical services^a^** | | | | | | | | |
| Days under lockdown^b^ | 0.94 | 0.82 | 1.09 | 0.429 | 1.08 | 1.01 | 1.14 | 0.016* |
| Age (years) | 0.97 | 0.88 | 1.07 | 0.596 | 1.04 | 0.99 | 1.10 | 0.085 |
| Sex (ref=male) | 1.56 | 1.16 | 2.11 | 0.004* | 1.66 | 1.44 | 1.91 | <0.001* |
| Household type (ref=multi-person household) | 1.08 | 0.70 | 1.65 | 0.738 | 1.12 | 0.89 | 1.40 | 0.356 |
| Marital status (ref=married/de facto) | 0.98 | 0.67 | 1.43 | 0.910 | 0.83 | 0.68 | 1.01 | 0.066 |
| Highest education achieved  (ref=tertiary or higher education) | 0.99 | 0.75 | 1.29 | 0.910 | 0.86 | 0.74 | 1.00 | 0.048* |
| Employment (ref=employed) | 1.20 | 0.87 | 1.65 | 0.277 | 1.17 | 0.99 | 1.38 | 0.074 |
| Long-term health conditions (ref=none) | 1.63 | 1.05 | 2.53 | 0.030* | 1.47 | 1.24 | 1.75 | <0.001* |
| PCS score^c^ | 1.31 | 1.11 | 1.54 | 0.001* | 1.24 | 1.14 | 1.34 | <0.001* |
| MCS score^d^ | 1.18 | 1.03 | 1.36 | 0.019* | 1.25 | 1.18 | 1.34 | <0.001* |
| **Disruption with dental services^a^** | | | | | | | | |
| Days under lockdown^b^ | 1.21 | 1.04 | 1.41 | 0.015* | 1.14 | 1.08 | 1.20 | <0.001* |
| Age (years) | 1.10 | 0.99 | 1.23 | 0.089 | 1.08 | 1.03 | 1.13 | 0.001* |
| Sex (ref=male) | 1.41 | 1.03 | 1.91 | 0.030* | 1.52 | 1.35 | 1.71 | <0.001* |
| Household type (ref=multi-person household) | 0.83 | 0.53 | 1.30 | 0.413 | 1.28 | 1.01 | 1.62 | 0.045* |
| Marital status (ref=married/de facto) | 1.23 | 0.82 | 1.84 | 0.315 | 0.75 | 0.61 | 0.92 | 0.006* |
| Highest education achieved  (ref=tertiary or higher education) | 0.71 | 0.52 | 0.98 | 0.035* | 0.64 | 0.56 | 0.74 | <0.001* |
| Employment (ref=employed) | 0.87 | 0.59 | 1.26 | 0.449 | 0.83 | 0.70 | 0.98 | 0.031* |
| Long-term health conditions (ref=none) | 1.24 | 0.79 | 1.93 | 0.349 | 1.15 | 0.97 | 1.36 | 0.111 |
| PCS score^c^ | 0.90 | 0.75 | 1.07 | 0.238 | 0.91 | 0.84 | 0.99 | 0.021* |
| MCS score^d^ | 1.06 | 0.90 | 1.24 | 0.487 | 0.98 | 0.92 | 1.05 | 0.541 |
| **Disruption with mental health providers^a^** | | | | | | | | |
| Days under lockdown^b^ | 1.33 | 1.06 | 1.67 | 0.015* | 1.07 | 0.94 | 1.21 | 0.319 |
| Age (years) | 0.61 | 0.50 | 0.74 | <0.001* | 0.76 | 0.69 | 0.84 | <0.001* |
| Sex (ref=male) | 1.61 | 0.81 | 3.20 | 0.175 | 1.30 | 0.95 | 1.77 | 0.102 |
| Household type (ref=multi-person household) | 1.12 | 0.49 | 2.55 | 0.781 | 0.77 | 0.49 | 1.22 | 0.269 |
| Marital status (ref=married/de facto) | 1.30 | 0.61 | 2.76 | 0.497 | 1.44 | 0.96 | 2.16 | 0.077 |
| Highest education achieved  (ref=tertiary or higher education) | 1.28 | 0.75 | 2.16 | 0.365 | 0.77 | 0.53 | 1.11 | 0.163 |
| Employment (ref=employed) | 2.08 | 1.00 | 4.33 | 0.051 | 1.31 | 0.95 | 1.79 | 0.096 |
| Long-term health conditions (ref=none) | 1.87 | 0.86 | 4.04 | 0.113 | 2.71 | 1.92 | 3.84 | <0.001* |
| PCS score^c^ | 1.24 | 0.89 | 1.74 | 0.201 | 1.00 | 0.85 | 1.18 | 0.996 |
| MCS score^d^ | 1.86 | 1.38 | 2.51 | <0.001* | 1.84 | 1.63 | 2.09 | <0.001* |
| **Disruption with allied health providers^a^** | | | | | | | | |
| Days under lockdown^b^ | 1.43 | 1.22 | 1.68 | <0.001* | 1.29 | 1.20 | 1.40 | <0.001* |
| Age (years) | 1.15 | 1.03 | 1.30 | 0.017* | 1.07 | 1.01 | 1.13 | 0.013* |
| Sex (ref=male) | 2.96 | 2.02 | 4.34 | <0.001* | 1.76 | 1.47 | 2.11 | <0.001* |
| Household type (ref=multi-person household) | 1.55 | 0.90 | 2.68 | 0.115 | 1.09 | 0.82 | 1.43 | 0.559 |
| Marital status (ref=married/de facto) | 0.89 | 0.55 | 1.43 | 0.622 | 0.89 | 0.69 | 1.14 | 0.353 |
| Highest education achieved  (ref=tertiary or higher education) | 0.65 | 0.46 | 0.91 | 0.013* | 0.60 | 0.51 | 0.72 | <0.001* |
| Employment (ref=employed) | 0.78 | 0.50 | 1.21 | 0.263 | 0.94 | 0.77 | 1.14 | 0.515 |
| Long-term health conditions (ref=none) | 1.59 | 1.01 | 2.52 | 0.048* | 1.62 | 1.33 | 1.96 | <0.001* |
| PCS score^c^ | 1.24 | 1.03 | 1.49 | 0.023* | 1.28 | 1.18 | 1.39 | <0.001* |
| MCS score^d^ | 1.12 | 0.94 | 1.33 | 0.197 | 1.08 | 0.99 | 1.18 | 0.074 |
| ^*^ p<0.05  ^a^ Logistic regression models for SES domains/subpopulations (lower SES and higher SES) included length of lockdown, age, sex, household type, marital status, highest education achieved, employment, long-term health conditions, PCS score and MCS score. Covariates included in the model (except for length of lockdown) were obtained from wave 19 (pre-pandemic)  ^b^ Incremental increase of 30 additional days of lockdown  ^c^ Incremental decrease of one SD in PCS score (1SD=10.69)  ^d^ Incremental decrease of one SD in MCS score (1SD=11.33)  Abbreviations: CI=Confidence interval; OR=Odds ratio (adjusted); MCS=Mental Component Summary; PCS=Physical Component Summary; ref=reference group; SD=Standard deviation; SES=Socio-economic status | | | | | | | | |

**Supplementary Figure 1. Physical and mental health quality-of-life trends from difference-in-differences models**

| **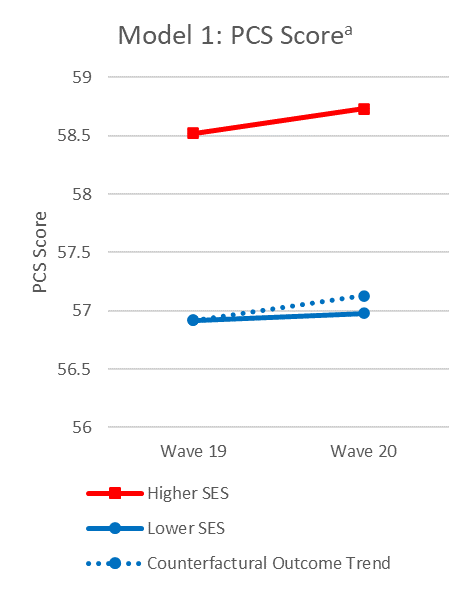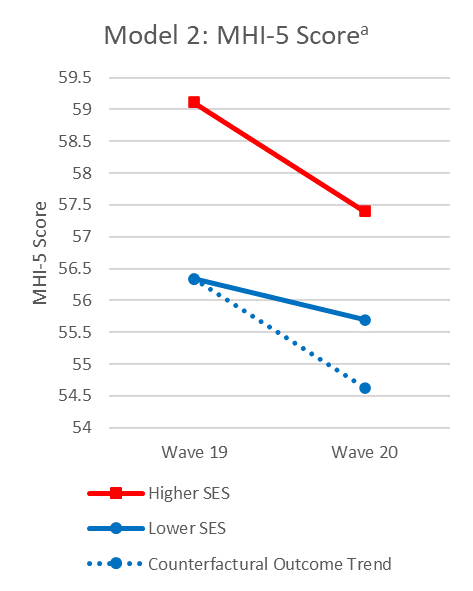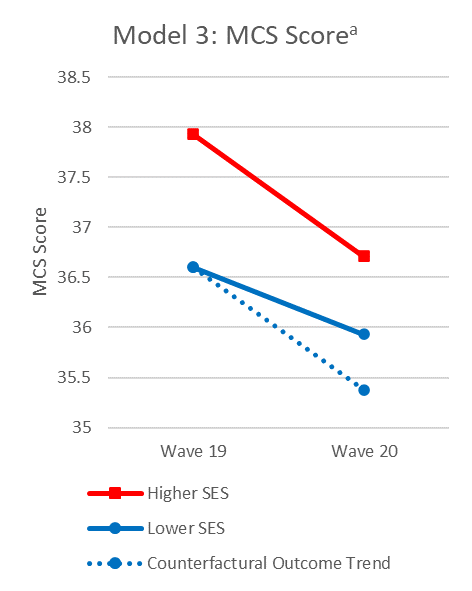**  Note: Difference-in-difference models for changes in quality-of-life measures (PCS, MHI-5, and MCS scores) between wave 19 (pre-pandemic) and 2020 (after the onset of the pandemic) by SES group. The counterfactual outcome trends show the unobserved trend for quality-of-life scores in the lower SES group if there was no difference in the changes in quality-of-life scores between the lower and higher SES groups.  ^a^ Higher scores indicate better physical health (PCS) or mental health (MHI-5 and MCS) quality-of-life.  Abbreviations: MCS=Mental Component Summary; MHI-5=Mental Health Inventory-5; PCS=Physical Component Summary; SES=Socio-economic status |
| --- |
